# Associations between gestational anthropometry, maternal HIV, and fetal and early infancy growth in a prospective rural/semi-rural Tanzanian cohort, 2012-13

**DOI:** 10.1186/s12884-015-0718-6

**Published:** 2015-10-29

**Authors:** Amanda L. Wilkinson, Sarah H. Pedersen, Mark Urassa, Denna Michael, Jim Todd, Safari Kinung’hi, John Changalucha, Joann M. McDermid

**Affiliations:** Division of Nutritional Sciences, Cornell University, Ithaca, NY USA; National Institute for Medical Research, Mwanza Research Centre, Mwanza, Tanzania; Department of Population Health, London School of Hygiene & Tropical Medicine, London, UK; Present affiliation: Division of Infectious Diseases & International Health, Department of Medicine, School of Medicine, University of Virginia, Charlottesville, VA USA

**Keywords:** Africa, HIV/AIDS, HIV-exposed, MUAC, Maternal, Nutrition, Point-of-care, Stunting, Triceps skinfold, Tanzania

## Abstract

**Background:**

Healthcare access and resources differ considerably between urban and rural settings making cross-setting generalizations difficult. In resource-restricted rural/semi-rural environments, identification of feasible screening tools is a priority. The objective of this study was to evaluate gestational anthropometry in relation to birth and infant growth in a rural/semi-rural Tanzanian prospective cohort of mothers and their infants.

**Methods:**

Mothers (*n* = 114: 44 HIV-positive) attending antenatal clinic visits were recruited in their second or third trimester between March and November, 2012, and followed with their infants through 6-months post-partum. Demographic, clinical, and infant feeding data were obtained using questionnaires administered by a Swahili-speaking research nurse on demographic, socioeconomic, clinical, and infant feeding practices. Second or third trimester anthropometry (mid-upper arm circumference [MUAC], triceps skinfold thickness, weight, height), pregnancy outcomes, birth (weight, length, head circumference) and infant anthropometry (weight-for-age z-score [WAZ], length-for-age z-score [LAZ]) were obtained. Linear regression and mixed effect modeling were used to evaluate gestational factors in relation to pregnancy and infant outcomes.

**Results and discussion:**

Gestational MUAC and maternal HIV status (HIV-positive mothers = 39 %) were associated with infant WAZ and LAZ from birth to 6-months in multivariate models, even after adjustment for infant feeding practices. The lowest gestational MUAC tertile was associated with lower WAZ throughout early infancy, as well as lower LAZ at 3 and 6-months. In linear mixed effects models through 6-months, each 1 cm increase in gestational MUAC was associated with a 0.11 increase in both WAZ (*P <* 0.001) and LAZ (*P =* 0.001). Infant HIV-exposure was negatively associated with WAZ (β = -0.65, *P <* 0.001) and LAZ (β = -0.49, *P <* 0.012) from birth to 6-months.

**Conclusions:**

Lower gestational MUAC, evaluated using only a tape measure and minimal training that is feasible in non-urban clinic and community settings, was associated with lower infant anthropometric measurements. In this rural and semi-rural setting, HIV-exposure was associated with poorer anthropometry through 6-months despite maternal antiretroviral access. Routine assessment of MUAC has the potential to identify at-risk women in need of additional health interventions designed to optimize pregnancy outcomes and infant growth. Further research is needed to establish gestational MUAC reference ranges and to define interventions that successfully improve MUAC during pregnancy.

## Background

Maternal malnutrition is an important risk factor for adverse infant outcomes, including low birth weight (LBW) [1]. Birth weight represents a key barometer of fetal health, and LBW infants are at increased risk of morbidity and mortality [2]. While maternal nutritional status is a useful predictor of pregnancy and birth outcomes [3], evaluating maternal nutritional status in resource-restricted regions remains challenging. Maternal height and weight are frequently unavailable, and if recorded, often inaccurate since adult weigh scales are costly to obtain and maintain calibrated. Clinical observation may be unreliable as maternal malnutrition can be masked by pregnancy-associated weight gain and fluid shifts.

Tanzania promotes the World Health Organization (WHO) antenatal care recommendations that include measuring weight gain, fundal height, hemoglobin, urinary albumin and glucose at antenatal visits, and measuring height plus screening for HIV and syphilis at least once [4]. While these indicators can identify severe maternal malnutrition and clinical complications, pregnant women with mild or moderate malnutrition may remain unidentified. Failure to recognize these women essentially eliminates any opportunity for them to receive additional monitoring and/or interventions that may prevent negative fetal and/or infant consequences. The workload burden of healthcare workers in this setting is considerable [5], and the identification of at-risk pregnancies represents the crucial first step towards achieving better maternal, fetal and infant care calls for quick, inexpensive, and feasible assessment – in essence, a point-of-care test (POCT).

Maternal MUAC measured during gestation has been shown to be associated with LBW in a number of African and Asian studies [6], although no reference ranges have been established. Despite widespread usage of MUAC to identify malnutrition in pediatric populations [7, 8], and to a lesser extent in pregnant and lactating women [9], gestational MUAC as a predictor of poor fetal or early infancy growth has not yet been recommended by WHO or any other public health agency. A non-governmental organization operating in humanitarian crisis settings, Médecins Sans Frontières, has recommended the use of maternal MUAC <23 cm to identify women at risk of delivering LBW infants based on the strength of the association between maternal MUAC and LBW and the simplicity of obtaining gestational MUAC measurements, without requiring knowledge of gestational age [10]. These same features suggest that maternal MUAC may be useful in non-urban health care and community settings in developing countries, and thus, the objective of this study was to investigate current Tanzanian standard antenatal (height, weight) and non-standard [MUAC, triceps skinfold thickness (TSF)] gestational anthropometric measurements in HIV-positive and HIV-negative women in association with birth and early infant growth in a prospective rural and semi-rural Tanzanian cohort.

## Methods

### Study setting and population

A prospective cohort of 44 HIV-positive and 70 HIV-negative pregnant women living in Magu District, Tanzania, was established between March to November 2012, with cohort observation continuing through July 2013. Participants were recruited from women seeking antenatal services at rurally-located dispensaries, representing the first level of public health care in Tanzania, and from women attending antenatal visits at Kisesa Health Centre, a publically accessible government-administrated healthcare facility located in the semi-rural region around Kisesa Village. Kisesa Health Centre offers basic antenatal and obstetrical services, outpatient child and adult healthcare, and provides HIV counselling, testing, and treatment. The clinic serves a large catchment area in the vicinity of Kisesa Village, located approximately 20 kilometers from the city of Mwanza. Participants in this observational study that were identified as malnourished or requiring additional clinical follow-up for any condition were referred via the study physician liaison to the main clinical services of Kisesa Health Centre. Mothers provided written informed consent for themselves and on behalf of their infants. Ethical approval for this study was granted by the Tanzanian National Health Research Ethics Review Committee and the Cornell University Institutional Review Board.

Study eligibility included stated intention to live within the antenatal clinic catchment area until 6-months postpartum, confirmed maternal HIV serostatus [screening by Determine™ HIV-1/2 (Inverness Medical), confirmation by Uni-Gold™ HIV-1/2 (Trinity Biotech)] at enrollment, and singleton pregnancy (any eligible pregnant woman was enrolled, but they were subsequently withdrawn if a multiple delivery was confirmed). All HIV-positive women who met the eligibility criteria were invited to participate and HIV-negative women were invited to participate in the study at approximately double the rate as HIV-negative women joined, although there was no specific matching strategy employed. The required sample sizes for statistical analyses assuming α = 0.05 (two-sided), power = 80 % and when applicable, unequal group size in the ratio = 2:1, varied according to the statistical procedure (e.g. Student’s *t*-test, Chi-squared test, adjusted and unadjusted linear regression) and cohort participants included (e.g. maternal analyses only, infant only, both). The sample size recruited exceeded that required to detect mean differences in birth weight (mean/standard deviation = 300/425 g), birth length (1.25/2.00 cm), head circumference (1.0 /1.5 cm), birth MUAC (0.75/2.00 cm), gestational age at birth (1.5/2.5 weeks). Detectable linear regression effect sizes were targeted between small to medium effects for unadjusted (by convention small to medium effect size set at 0.02 < f < 0.15) to medium to large (by convention medium to large effect size set at 0.15 < f < 0.35) for adjusted (one predictor, six covariates) regression models.

Women were enrolled in the cohort after completing their first trimester. A follow-up visit was scheduled during pregnancy if the original enrollment date preceded the anticipated delivery date by ≥3 weeks. All women were followed-up at delivery if they delivered at Kisesa Health Centre or within 72 h if they delivered elsewhere. Thereafter, women were followed with their infants at 1, 2, 3, and 6-months postpartum. Mother-infant follow-up visits corresponded to Tanzanian infant vaccination visits, with the exception of a study-specific visit at 6-months. If a mother-infant pair failed to return for a follow-up visit, a field worker traveled to their last known address to invite them to return to the clinic for a rescheduled appointment. Recruitment and retention strategies included compensating participants for transportation expenses as region-specific surveys indicated transportation expenses were a significant barrier to accessing clinic-based services in this region [11].

**Fig. 1:**
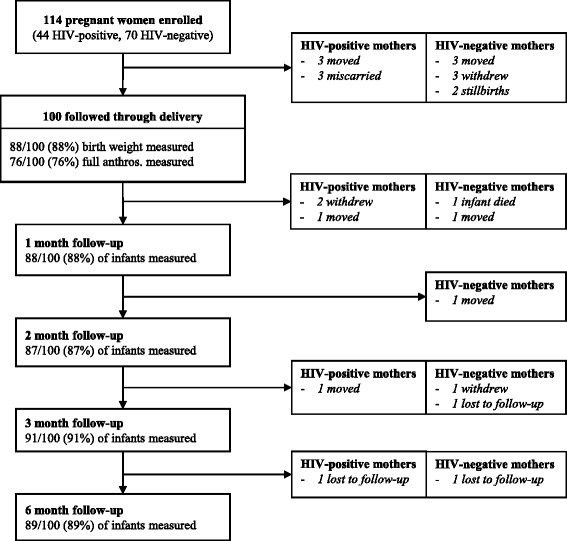
Maternal and infant cohort follow-up

Maternal HIV testing is offered as part of routine antenatal care in Tanzania and many women are first diagnosed with HIV infection during pregnancy. At the time of this study, Tanzania followed Option A of the WHO 2010 prevention of mother-to-child transmission (PMTCT) guidelines [12]. Women with absolute CD4 cell counts ≤350 cells/μL or WHO clinical stage 3 or 4 irrespective of CD4 cell count were eligible for a first-line combination antiretroviral regimen, consisting of zidovudine (AZT) + lamivudine (3TC) + efavirenz (EFV). All other HIV-positive women received AZT for PMTCT starting as early as 14 weeks gestation. HIV-exposed infants were prescribed daily nevirapine for six weeks, followed by HIV testing at 3-months of age using dried blood spot HIV DNA-PCR that was analyzed at Bugando Medical Centre in Mwanza City, the closest regional hospital laboratory. Out of the 38 HIV-exposed infants enrolled in the cohort at delivery, 32 (84 %) tested negative for HIV at 3-months and 6 (16 %) exited the study prior to HIV testing (Fig. 1).

### Data collection

Demographic, medical history, and infant feeding data were obtained through questionnaires administered by a Swahili-speaking research nurse. In the absence of ultrasound technology, participants were counseled to estimate their last menstrual period from which gestational age was approximated, or for the 6 % (*n* = 7/114) of women unable to estimate their last menstrual period, gestational age was estimated from the first available fundal height according to standard clinic procedures. All maternal and infant anthropometric measurements were collected by trained research nurses. Gestational anthropometric measurements were collected upon enrollment that corresponded to the second or third trimester of pregnancy, and included weight, height, MUAC, and TSF. Maternal weight and height were measured using a standard balance beam scale with a height rod (Health O Meter, Inc., Bridgeview, IL) to the precision of 0.2 kg and 0.1 cm, respectively. MUAC was measured by tape measure to the nearest 0.1 cm and TSF was measured by Lange Skin Calipers (Cambridge Scientific Industries, Cambridge, MD) to the nearest 0.1 mm. Maternal blood was drawn for hemoglobin quantification and anemia was defined as hemoglobin (Hb) concentration <11 g/dL and severe anemia as Hb <8.5 g/dL according to reference values used for clinical referral in Tanzania [13]. All women identified as anemic were referred for clinical follow-up at Kisesa Health Centre. Infant birth anthropometric measurements (weight, height, head circumference, MUAC) were included in the birth anthropometry analyses if collected within 72 h of delivery (Fig. 1). Infant anthropometry was measured at 1, 2, 3 and 6-months. Infant weight and length were measured using a digital infant scale (Seca 334 Digital Baby Scale) to the nearest 0.01 kg and 0.1 cm, respectively, and infant tape measures were used to measure both infant head circumference and MUAC to the nearest 0.1 cm.

Exclusive breastfeeding was defined according to the WHO definition, which allows for the infant to receive only breast milk and medications, oral rehydration solutions, vitamins and minerals [14]. Exclusive breastfeeding duration was defined as the period between birth and the age at which the infant first received food or non-breast milk liquids, other than medications. A study-specific breastfeeding score was evaluated at each visit, and the individual visit scores were summed to provide an overall breastfeeding score. Infant feeding practices were scored as: 0 = no breastfeeding; 1 = partial breastfeeding (defined as breast milk plus other foods and/or liquids); 2 = predominant breastfeeding (defined as breast milk plus locally-prepared non-prescription anti-colic/anti-gripe water); 3 = exclusive breastfeeding. The overall breastfeeding score summarized infant feeding practices over the one to 6-month period, with scores ranging from 0 to 12. An example score for an infant exclusively breastfed for 6-months would include a visit-specific breastfeeding score of 3 at each of 4 follow-up visits = 12. An example score for an infant who was exclusively breastfeeding at month-1, transitioned to predominant breastfeeding at month-2, transitioned to partial breastfeeding at month-3 and stopped all breastfeeding by the month-6 visit would be 3 + 2 + 1 + 0 = 6. If a mother missed a follow-up visit, information regarding infant feeding practices for the relevant month was ascertained at the subsequent study visit.

### Statistical analysis

Data were analyzed in STATA12 (STATA Corporation, Texas, USA). Means of normally-distributed continuous variables were compared using Student’s *t-*test and one-way analysis of variance (ANOVA) was used for comparisons of means across categorical variables. Proportions of categorical variables were compared using Pearson chi-square tests. Relationships between infant anthropometrics at birth and gestational clinical characteristics were analyzed using both unadjusted linear regression models, and linear regression models adjusted for covariates chosen *a priori* (maternal HIV status, age, parity, education, gestational age at the time of anthropometric measurement, and infant sex). Gestational MUAC was examined as both a continuous and categorical variable classified according to study-specific data-derived tertiles because there are no established gestational MUAC reference values. Infant weight-for-age (WAZ) and length-for-age (LAZ) z-scores were calculated using the WHO Child Growth Standards with the *igrowup* macro package (freely available at: www.who.int/childgrowth/software/en). Underweight and stunting were defined as having a z-score < -2 standard deviations below the WHO reference standards for WAZ and LAZ, respectively. Mixed effect models were used to compare infant growth trajectories between groups (HIV-exposed vs. HIV-unexposed and low vs. high gestational MUAC). For these analyses, MUAC was dichotomized into “low” and “high”, with low MUAC defined as MUAC is the lowest tertile of the cohort distribution and high MUAC defined as MUAC in the middle and highest tertiles combined. Posthoc pairwise comparisons were made at each age corresponding to study visit (birth, 1, 2, 3, and 6-months) and were based on estimated margins means. To account for multiple comparisons, the Bonferroni correction procedure was used in these analyses. Mixed effect models were also used to examine the relationships between infant anthropometry and covariates of interest (HIV and gestational MUAC), with adjustment for potential confounding variables.

## Results

### Maternal characteristics

Characteristics of the 44 HIV-positive and 70 HIV-negative women enrolled in the study and their infants are summarized in Table 1. Mean BMI did not differ significantly by maternal HIV status. Gestational MUAC (tertile) was positively associated with gestational TSF, weight, and BMI (for all, ANOVA *P <* 0.001). The average gestational age was 27 weeks at study enrollment (range: 13-39 weeks), with HIV-positive women enrolling significantly earlier in pregnancy. Most women were married (87 %) and multiparous (86 %), and while the majority (89 %) of women reported being formally educated, only 11 % advanced beyond the primary school level. Few households had electricity (10 %), and many women worked to generate household income (78 %).

**Table 1:** Characteristics of pregnant women and their newborn infants in a prospective cohort, Tanzania, 2012-2013^a^

		Maternal HIV status			Gestational MUAC at enrollment (cm)			
Characteristics	Overall	Positive	Negative	*P*	≤25.5	25.6-27.9	≥28.0	*P*
	(*n* = 114)	(*n* = 44)	(*n* = 70)		(*n* = 42)	(*n* = 35)	(*n =* 37)	
Maternal characteristics at enrollment								
Gestational age at enrollment, weeks	26.9 ± 6.9	23.7 ± 7.1	28.9 ± 6.0	<0.001^b^	28.5 ± 5.8	25.4 ± 7.2	26.4 ± 7.4	0.135^c^
Age, y	28.0 ± 6.0	28.9 ± 6.0	27.4 ± 6.0	0.192^b^	27.4 ± 5.8	27.2 ± 5.6	29.4 ± 6.5	0.202^c^
Married, %	86.8	72.7	95.7	<0.001^d^	85.7	91.4	83.8	0.608^d^
Primiparous, %	14.0	15.9	12.9	0.648^d^	16.7	14.3	10.8	0.755^d^
Mid-upper arm circumference, cm	26.9 ± 2.7	27.3 ± 2.9	26.6 ± 2.5	0.192^b^	24.2 ± 1.2^a^	26.9 ± 0.6^b^	29.9 ± 1.6^c^	<0.001^c^
Triceps skinfold thickness, mm	14.1 ± 5.4	14.6 ± 5.1	13.9 ± 5.5	0.499^b^	10.2 ± 2.6^a^	13.6 ± 3.8^b^	19.1 ± 5.1^c^	<0.001^c^
Weight, kg	60.0 ± 7.8	60.3 ± 8.2	60.0 ± 7.6	0.859^b^	54.4 ± 5.2^a^	59.0 ± 5.1^b^	67.2 ± 6.8^c^	<0.001^c^
Height, cm	160.0 ± 5.5	160.1 ± 5.3	159.8 ± 5.7	0.788^b^	159.3 ± 5.3	160.3 ± 5.9	160.4 ± 5.6	0.611^c^
Body mass index, kg/m^b^	23.4 ± 2.6	23.4 ± 2.9	23.4 ± 2.5	0.928^b^	21.4 ± 1.4^a^	23.0 ± 1.8^b^	26.1 ± 2.0^c^	<0.001^c^
HIV-positive, %	38.6	100.0	--	--	33.3	34.3	48.7	0.310^d^
CD4 cell count, cells/μL (*n =* 43)^*5*^	--	560 ± 290	--	--	568 ± 272	535 ± 274	572 ± 326	0.939^c^
<200 cells/μL (*n =* 4),%	--	9.3	--	--	7.7	16.7	5.6	0.194^d^
200 to 349 (*n =* 7), %	--	16.3	--	--	7.7	0.0	33.3	--
350 to 499 (*n =* 12), %	--	27.9	--	--	30.8	41.7	16.7	--
≥500 (*n =* 20), %	--	46.5	--	--	53.9	41.7	44.4	--
	(*n* = 100)	(*n* = 38)	(*n* = 62)		(*n* = 39)	(*n* = 29)	(*n* = 32)	
Infant characteristics at birth^f^								
Gestational age at birth, weeks (*n =* 100)	38.2 ± 3.4	37.7 ± 3.0	38.4 ± 3.6	0.274^b^	37.6 ± 3.8	37.9 ± 3.3	39.0 ± 2.9	0.198^c^
Preterm birth (<37 weeks), %	30.0	34.2	27.4	--	30.8	31.0	28.1	--
Birth weight, g (*n =* 88)	3183 ± 448	3039 ± 493	3274 ± 396	0.016^b^	3064 ± 440	3209 ± 456	3290 ± 434	0.124^c^
Low birth weight (<2500 g), %	4.6	5.9	3.7	--	6.1	8.3	0.0	--
Birth length, cm (*n =* 76)	46.7 ± 2.0	46.0 ± 2.4	47.1 ± 1.6	0.026^b^	46.1 ± 1.9^a^	46.4 ± 2.2^a,b^	47.4 ± 1.8^b^	0.036^c^
Birth mid-upper arm circumference, cm (*n =* 76)	10.7 ± 1.1	10.6 ± 1.1	10.8 ± 1.0	0.397^b^	10.2 ± 2.2	9.6 ± 3.3	10.3 ± 2.9	0.694^c^
Birth head circumference, cm (*n* = 76)	34.6 ± 1.6	34.2 ± 1.4	34.7 ± 1.7	0.183^b^	34.0 ± 1.4	35.1 ± 1.7	34.7 ± 1.7	0.056^c^

Abbreviations: *HIV* human immunodeficiency virus, *MUAC* mid-upper arm circumference. The “--” symbol indicates data that are not applicable

^a^Values are means ± standard deviation or frequencies

^b^Student’s *t*-test for comparisons of means between groups based on maternal HIV status as HIV-positive vs HIV-negative mothers

^c^One-way ANOVA test for comparisons of means between groups based on gestational MUAC values of MUAC = ≤25.5 vs 25.6 to 27.9 vs ≥28.0 cm; for significant results, post hoc comparisons were made. Different superscript letters denote significant differences (*P* < 0.05) between the groups

^d^Pearson chi-square test for comparison of proportions

^e^CD4 cell count available from HIV-positive women only; CD4 was missing from one HIV-positive participant

^f^All birth anthropometric measurements were obtained within 72 h of birth. Birth data were available from 100/114 of the original cohort participants as 5/114 of pregnancies ended in miscarriage or stillbirth and 9/114 women exited the study prior to giving birth

Overall, hemoglobin concentration was low (mean ± standard deviation: 10.1 ± 1.8 g/dL), and anemia common (55 %, Hb <11.0 g/dL), with 11 % severely anemic (Hb <8.5 g/dL). Neither anemia nor severe anemia frequencies differed according to maternal HIV status (*P =* 0.494; *P =* 0.980, respectively), and HIV-positive women were not statistically different from HIV-negative women in their mean anthropometric indicators. Among HIV-positive women, advanced immunosuppression was rare as 3/4 had CD4 ≥ 350 cells/μL and almost half (46.5 %) had normal absolute CD4 cell counts (≥500 cells/μL). At enrollment, ART regimens included: 1) no ART (*n* = 5; 11 %); 2) triple therapy (AZT + 3TC + EFV; *n* = 15; 34 %) or 3) AZT monotherapy (*n* = 24; 55 %). Of the five women who were not receiving ART at enrollment, three initiated AZT before their next antenatal visit, one withdrew from the study, and one miscarried. All women who were taking triple therapy reported initiating this regimen for their own HIV care prior to becoming pregnant. Among those women who were prescribed AZT for PMTCT during pregnancy, 11 % initiated AZT during their first trimester, 79 % in their second and 11 % during their third trimester. By delivery, all HIV-positive women were receiving either triple therapy for their own HIV management, or AZT for PMTCT.

### Pregnancy outcomes and birth anthropometrics

In this rural and semi-rural cohort, 5 % of pregnancies ended in stillbirth or miscarriage, almost 1/3 delivered preterm, and 5 % of infants were born LBW (Table 1). HIV-positive mothers experienced a greater proportion of adverse outcomes (combined fetal death, preterm delivery, LBW: 41 vs 31 %, *P =* 0.29), and delivered babies with lower average birth weight and length. Infant birth weight was available from 89 % (34/38) of HIV-positive mothers and 87 % (54/62) of HIV-negative mothers who remained in the cohort through delivery (*P =* 0.72 for comparison) and complete infant birth anthropometric data were available from 76 % (29/38) of HIV-positive mothers and 76 % (47/62) of HIV-negative mothers (*P =* 0.95). Decreasing gestational MUAC tertiles were associated with decreasing birth length (ANOVA *P =* 0.036; Table 2). In adjusted regression models (Table 3), maternal HIV-seropositivity was associated with lower mean birth weight (-280 g; *P =* 0.013) and length (-1.03 cm; *P =* 0.052).

**Table 2:** Unadjusted linear regression of infant birth outcomes with maternal HIV status and nutritional status at enrollment

Main predictor	Gestational age at birth, wk (*n* = 100)			Birth weight, g (*n* = 88)			Birth length, cm (*n* = 76)			Birth MUAC, cm (*n* = 76)			Birth head circumference, cm (*n* = 76)		
	β	*P*	95 % CI	β	*P*	95 % CI	β	*P*	95 % CI	β	*P*	95 % CI	β	*P*	95 % CI
MUAC, cm	0.21	0.09	-0.04, 0.46	26.11	0.13	-8.10, 60.32	0.18	0.03	0.02, 0.35	0.06	0.16	-0.02, 0.15	0.05	0.45	-0.09, 0.19
TSF, mm	0.08	0.23	-0.05, 0.20	9.30	8.56	-7.71, 26.32	0.09	0.04	0.01, 0.17	0.03	0.13	-0.01, 0.08	0.04	0.25	-0.03, 0.11
Weight, kg	0.02	0.70	-0.07, 0.10	11.69	0.04	0.33, 23.05	0.06	0.03	0.00, 0.11	0.03	0.05	0.00, 0.06	0.01	0.59	-0.03, 0.06
Height, cm	-0.03	0.61	-0.15, 0.09	8.80	0.31	-8.26, 25.85	0.03	0.46	-0.05, 0.11	0.01	0.50	-0.03, 0.06	-0.01	0.79	-0.08, 0.06
BMI, kg/m^2^	0.10	0.13	-0.15, 0.35	29.07	0.10	-5.60, 63.73	0.18	0.03	0.01, 0.35	0.09	0.05	-0.00, 0.18	0.05	0.46	-0.09, 0.19
HIV-seropositivity	-0.77	0.27	-2.16, 0.62	-234.53	0.02	-424.07, -44.99	-1.04	0.03	-1.95, -0.13	-0.25	0.70	-1.52, 1.03	-0.51	0.18	-1.28, 0.25
	M*e*an ± SD		*P*	Mean ± SD		*P*	Mean ± SD		*P*	Mean ± SD		*P*	Mean ± SD		*P*
MUAC tertile^a^ (MUAC range)															
Tertile 1 (20.7-25.5 cm)	37.6 ± 0.5		0.198	3064 ± 77		0.124	46.1 ± 0.4^a^		0.036	10.5 ± 0.2		0.208	34.0 ± 0.3		0.056
Tertile 2 (25.7-27.8 cm)	37.9 ± 0.6			3209 ± 90			46.4 ± 0.4^a,b^			10.6 ± 0.2			35.1 ± 0.4		
Tertile 3 (28.0-33.5 cm)	39.0 ± 0.6			3290 ± 79			47.4 ± 0.4^b^			11.0 ± 0.2			34.7 ± 0.3		

Abbreviations: *HIV* human immunodeficiency virus, *MUAC* mid-upper arm circumference, *HC* head circumference, *BMI* body mass index, *TSF* triceps skinfold thickness

^a^Data are predicted means and standard deviations: for significant results, post hoc comparisons were made. Groups with different subscript letters are significantly different (*P* < 0.05)

**Table 3:** Adjusted linear regression of infant birth outcomes with maternal HIV status and nutrition at enrollment

Main predictor	Gestational age at birth, wk (*n* = 100)			Birth weight, g (*n* = 88)			Birth length, cm (*n* = 76)			Birth MUAC, cm (*n* = 76)			Birth head circumference, cm (*n* = 76)		
	β	*P*	95 % CI	β	*P*	95 % CI	β	*P*	95 % CI	β	*P*	95 % CI	Β	*P*	95 % CI
MUAC, cm	0.24	0.06	-0.01, 0.50	27.44	0.12	-7.26, 62.15	0.21	0.01	0.04, 0.38	0.06	0.23	-0.04, 0.15	0.08	0.31	-0.07, 0.22
TSF, mm	0.08	0.24	-0.05, 0.21	10.56	0.24	-7.11, 28.23	0.09	0.04	0.01, 0.17	0.03	0.19	-0.02, 0.08	0.05	0.14	-0.02, 0.13
Weight, kg	0.01	0.89	-0.08, 0.09	13.89	0.02	2.50, 25.28	0.06	0.04	0.00, 0.11	0.03	0.04	0.00, 0.06	0.01	0.53	-0.03, 0.06
Height, cm	-0.03	0.62	-0.16, 0.09	12.71	0.14	-4.37, 29.78	0.04	0.30	-0.04, 0.13	0.02	0.35	-0.02, 0.07	-0.00	0.98	-0.07, 0.07
BMI, kg/m^b^	0.06	0.66	-0.20, 0.31	31.41	0.08	-3.32, 66.14	0.16	0.06	-0.01, 0.33	0.09	0.06	-0.00, 0.18	0.05	0.48	-0.09, 0.20
HIV-seropositivity	-0.18	0.82	-1.74, 1.38	-280.38	0.01	-500.80, -59.96	-1.03	0.05	-2.08, 0.01	-0.21	0.78	-1.66, 1.25	-0.41	0.36	-1.29, 0.48
	Mean ± SD		*P*	Mean ± SD		*P*	Mean ± SD		*P*	Mean ± SD		*P*	Mean ± SD		*P*
MUAC tertile^b^ (MUAC range)															
Tertile 1 (20.7-25.5 cm)	37.5 ± 0.5		0.159	3062 ± 78		0.119	46.0 ± 0.4^a^		0.016	10.7 ± 0.9		0.252	33.9 ± 0.3^a^		0.025
Tertile 2 (25.7-27.8 cm)	38.0 ± 0.6			3185 ± 91			46.4 ± 0.4^a,b^			10.6 ± 0.9			35.2 ± 0.4^b^		
Tertile 3 (28.0-33.5 cm)	39.1 ± 0.6			3311 ± 81			47.5 ± 0.4^b^			11.1 ± 0.9			34.8 ± 0.3^a,b^		

^a^All models have been adjusted for maternal age, parity, education, gestational age at the time of gestational anthropometric measurement, and infant gender. Models with gestational nutritional indicators as the main predictor have also been adjusted for maternal HIV status. Abbreviations: *HIV* human immunodeficiency virus, *MUAC* mid-upper arm circumference, *HC* head circumference, *BMI* body mass index, *TSF* triceps skinfold thickness

^b^Data are predicted means and standard deviations, adjusted for the covariates listed in footnote 1; for significant results, post hoc comparisons were made. Groups with different subscript letters are significantly different (*P* < 0.05)

Gestational MUAC, TSF, and BMI at enrollment were each associated with greater infant birth length, and maternal weight during pregnancy was positively associated with birth weight and length. Gestational MUAC (tertile) remained significantly associated with birth length in adjusted analyses (Table 3). In the adjusted analyses of birth outcomes, interactions between gestational MUAC and HIV were examined, but were not statistically significant (interaction p-values: gestational age at delivery, *P =* 0.231; birth weight *P =* 0.446; birth length, *P =* 0.474; birth MUAC, *P =* 0.602; birth head circumference, *P* = 0.075).

### Infant growth

**Fig. 2:**
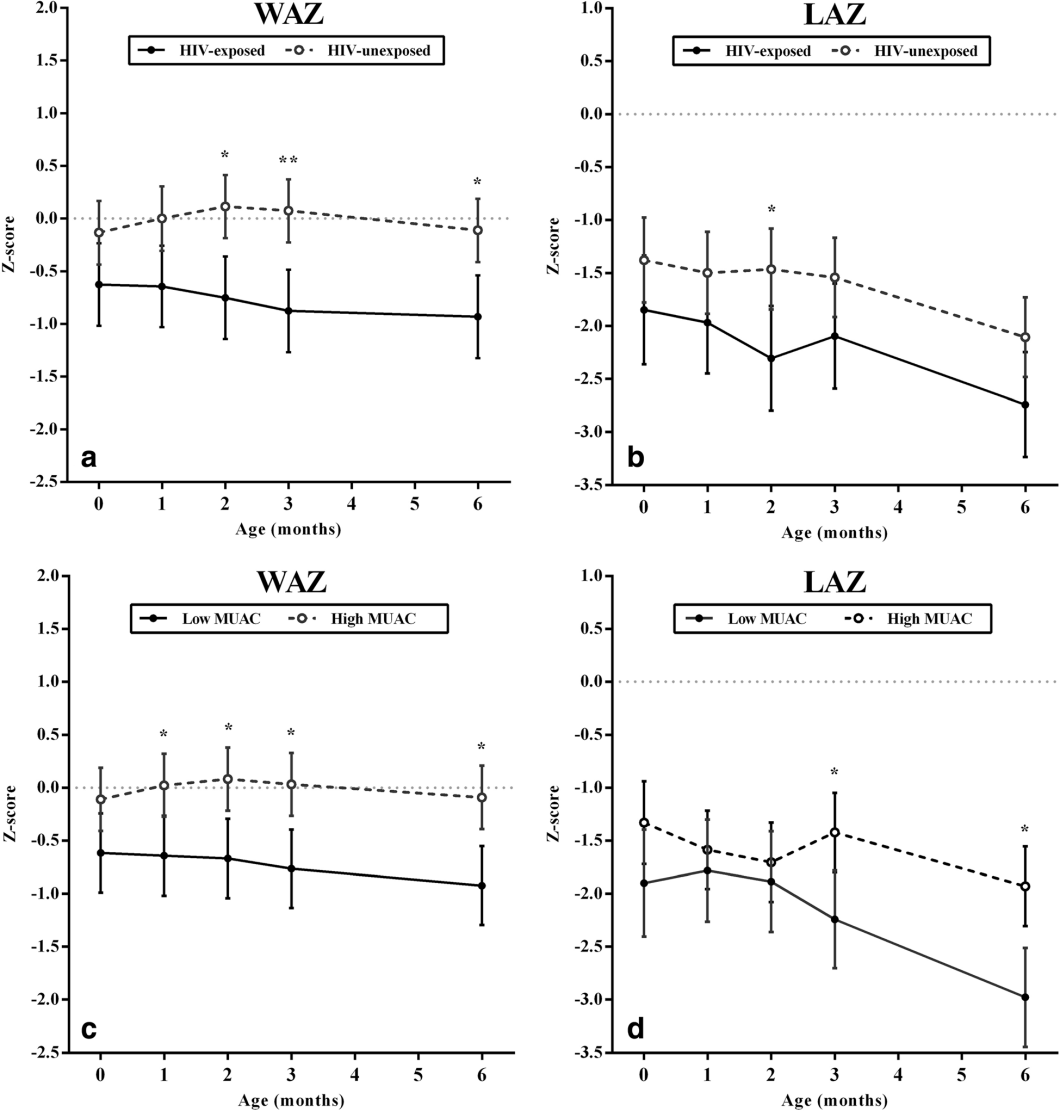
Mean weight-for-age and length-for-age z-scores, stratified on infant HIV-exposure or gestational mid-upper arm circumference. Models stratified on infant HIV-exposure (**a** and **b**, where “HIV-U” = infant HIV-unexposed, “HIV-E” = infant HIV-exposed) or gestational mid-upper arm circumference (**c** and **d**, where “Low MUAC” = lowest gestational mid-upper arm circumference tertile, “High MUAC” = middle and highest tertiles combined). Dotted lines depict z-scores of zero. All models adjusted for gestational age; post hoc pairwise comparisons used to generate predicted marginal means and 95 % CI at each time point. *P < 0.01, **P < 0.001

**Fig. 3:**
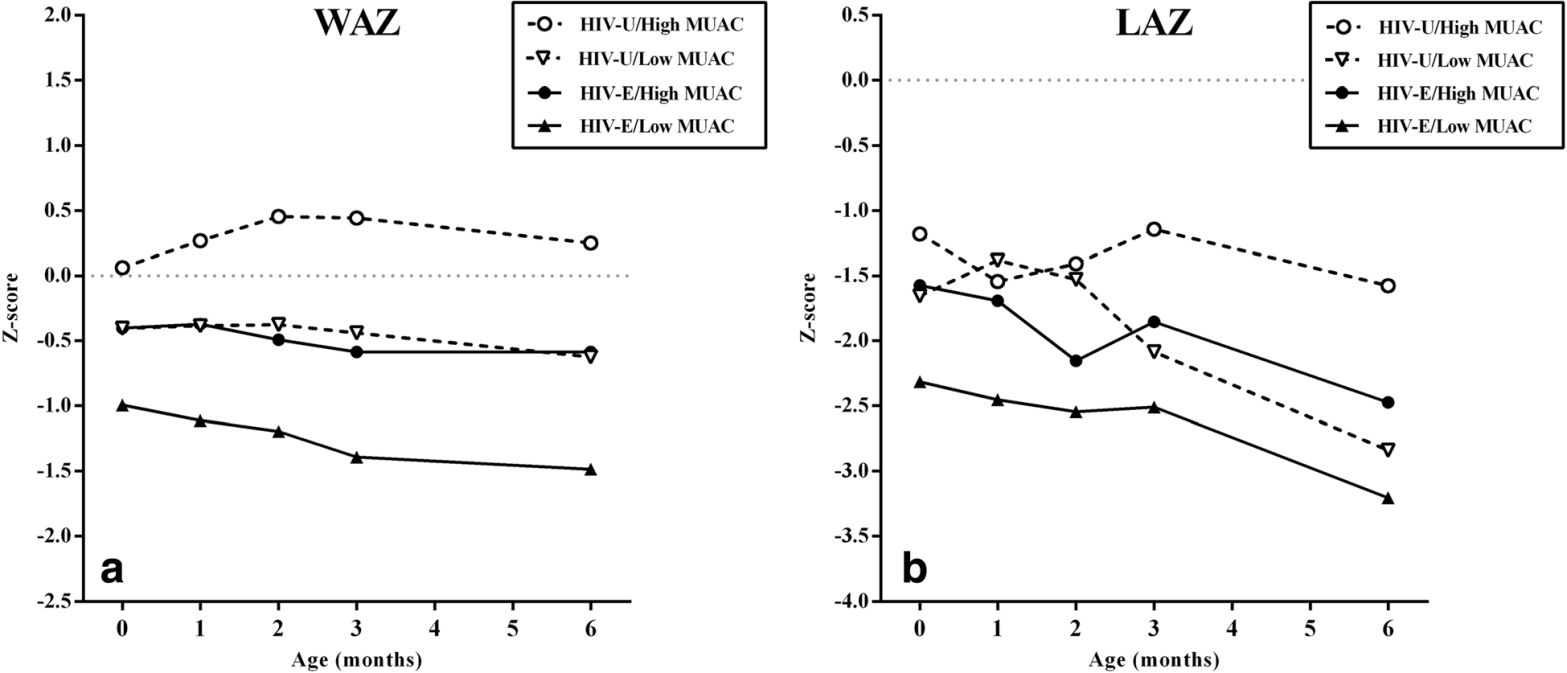
Mean weight-for-age and length-for-age z-scores, stratified on infant HIV-exposure plus gestational mid-upper arm circumference. Models stratified on infant HIV-exposure (Fig. 2a and b, where “HIV-U” = infant HIV-unexposed, “HIV-E” = infant HIV-exposed) or gestational mid-upper arm circumference (Fig. 2c and d, where “Low MUAC” = lowest gestational mid-upper arm circumference tertile, “High MUAC” = middle and highest tertiles combined). Dotted lines depict z-scores of zero. All models adjusted for gestational age; post hoc pairwise comparisons were used to generate predicted marginal means and 95 % CI at each time point and the significance threshold after Bonferroni correction; significance was *P* < 0.01. Significant differences were observed for the following weight-for-age z-scores: -HIV-U/High MUAC vs. HIV-U/Low MUAC at months 2, 3, and 6; -HIV-U/High MUAC vs. HIV-E/High MUAC at months 2 and 3; -HIV-U/High MUAC vs. HIV-E/Low MUAC at each time points. Significant differences were observed for the following length-for-age z-scores: -HIV-U/High MUAC vs. HIV-U/Low MUAC at month 6; -HIV-U/High MUAC vs. HIV-E/Low MUAC at months 3 and 6

Unadjusted infant anthropometry data is summarized in Table 4. In this rural and semi-rural cohort, the number of infants classified as stunted increased throughout early infancy with the majority being stunted by 6-months (57 %, n = 51). HIV-exposed infants were disproportionally affected by underweight and stunting throughout the first 6-months. In growth models adjusted for gestational age at enrollment, HIV-exposed infants had significantly lower WAZ at 2, 3 and 6-months and lower LAZ at 2-months (Fig. 2a-b). Infant growth differences based on HIV-exposure persisted, despite HIV-positive women reporting a longer mean duration of exclusive breastfeeding (HIV-positive: 6.6 ± 1.4 weeks vs. HIV-negative: 3.4 ± 0.7 weeks; *P* = 0.03) and a significantly higher breastfeeding score (HIV-positive: 7.5 ± 0.4 vs. HIV-negative: 6.1 ± 0.3; *P* = 0.004). Infant growth outcomes also differed according to gestational MUAC. “Low” gestational MUAC (corresponding to the lowest tertile of the cohort distribution and a MUAC of ≤25.5 cm) was associated with significantly lower WAZ throughout infancy and lower LAZ at 3 and 6-months compared to high gestational MUAC (corresponding to the middle and highest tertiles combined, and a MUAC >25.5 cm) (Fig. 2c-d). Infant feeding practices did not differ according to gestational MUAC (low vs. high MUAC comparisons; exclusive breastfeeding duration, *P =* 0.539; breastfeeding score, *P =* 0.377). Among the infants in this cohort, those with dual adverse maternal exposures (HIV-positive mothers with gestational MUAC in the lowest terile) had the poorest growth from birth through 6-months based on both WAZ and LAZ (Fig. 3). Mixed effect models of infant growth adjusted for potential confounding factors confirm the significant association between maternal HIV and maternal nutritional status according to gestational MUAC with early infant growth outcomes (Table 5). MUAC, modeled as a continuous variable, was also significantly associated with infant WAZ and LAZ, with each 1 cm increase in maternal MUAC associated with a +0.11 (*P* < 0.001) increase in infant WAZ, as well as a +0.11 (*P =* 0.001) increase in infant LAZ. Infant HIV-exposure had statistically significant, negative associations with WAZ (β = -0.65, *P <* 0.001) and LAZ (β = -0.49, *P <* 0.012) from birth to 6-months in mixed effect models. Again, interactions were examined between maternal HIV and gestational MUAC in these growth models, but they were not statistically significant (interaction p-values: WAZ *P =* 0.138; LAZ *P =* 0.525).

**Table 4:** Early infancy anthropometry, stratified by HIV exposure and gestational mid-upper arm circumference at enrollment^a^

	Age	0 (birth)	1-month	2-months	3-months	6-months
Overall	N	88	88	87	91	89
	Weight-for-age z-score	-0.27 (-0.48, -0.52)	-0.23 (-0.47, -0.00)	-0.15 (-0.42, 0.12)	-0.28 (-0.55, 0.01)	-0.41 (-0.68, -0.13)
	Length-for-age z-score	-1.52 (-1.76, -1.28)^b^	-1.70 (-2.01, -1.38)	-1.77 (-2.09, -1.44)	-1.75 (-2.05, -1.45)	-2.35 (-2.70, -1.99)
	Underweight, %	3.4	4.6	8.1	8.8	6.7
	Stunting, %	25.0	44.3	48.3	41.1	57.3
HIV-exposed	N	34	36	33	34	33
	Weight-for-age z-score	-0.61 (-1.00, -0.22)*	-0.63 (-1.06, -0.20)**	-0.67 (-1.16, -0.18)**	-0.86 (-1.34, -0.38)***	-0.88 (-1.40, -0.36)**
	Length-for-age z-score	-1.89 (-2.39, -1.40)*^b^	-1.99 (-2.53, -1.44)	-2.27 (-2.85, -1.70)*	-2.10 (-2.63, -1.56)	-2.71 (-3.27, -2.15)
	Underweight,%	5.9	11.1*	18.2**	17.7*	15.2*
	Stunting, %	37.9*	55.6	63.6*	48.9	66.7
HIV-unexposed	N	54	52	54	57	56
	Weight-for-age z-score	-0.05 (-0.29, 0.19)	0.04 (-0.20, 0.28)	0.17 (-0.13, 0.46)	0.06 (-0.24, 0.36)	-0.13 (-0.42, 0.17)
	Length-for-age z-score	-1.29 (-1.53, -1.05)^b^	-1.49 (-1.87, -1.11)	-1.46 (-1.84, -1.07)	-1.55 (-1.91, -1.19)	-2.13 (-2.60, -1.67)
	Underweight, %	1.9	0.0	1.9	3.5	1.8
	Stunting, %	17.0	36.5	38.9	36.8	51.8
Low gestational mid-upper arm circumference	N	33	32	33	36	35
	Weight-for-age z-score	-0.53 (-0.90, -0.15)	-0.65 (-1.04, -0.27)**	-0.53 (-0.96, -0.11)*	-0.75 (-1.14, -0.36)**	-0.86 (-1.23, -0.49)**
	Length-for-age z-score	-1.81 (-2.20, -1.43)^b^	-1.83 (-2.34, -1.32)	-1.82 (-2.36, -1.28)	-2.21 (-2.64, -1.77)	-2.93 (-3.60, -2.26)**
	Underweight, %	6.1	6.3	9.1	11.1	8.6
	Stunting, %	32.1	43.8	48.5	52.8	71.4*
High gestational mid-upper arm circumference	N	55	56	54	55	54
	Weight-for-age z-score	-0.11 (-0.37, 0.15)	0.01 (-0.27, 0.28)	0.09 (-0.26, 0.43)	0.02 (-0.33, 0.38)	-0.11 (-0.48, 0.26)
	Length-for-age z-score	-1.35 (-1.66, -1.04)^b^	-1.62 (-2.03, -1.21)	-1.73 (-2.16, -1.31)	-1.45 (-1.84, -1.06)	-1.97 (-2.35, -1.58)
	Underweight, %	1.8	3.6	7.4	7.3	5.6
	Stunting, %	20.8	44.6	48.2	33.3	48.2

^a^Data are unadjusted means and 95 % confidence intervals. “Low” gestational mid-upper arm circumference was classified as the lowest tertile of the cohort distribution (range: 20.7-25.5 cm) and high as the middle and highest tertiles combined (range: 25.7-33.5 cm). Significant differences between groups (HIV-exposed vs. HIV-unexposed; low vs. high gestational mid-upper arm circumference at enrollment) were tested using Student’s *t*-test for continuous variable and Pearson chi-square test for categorical outcomes (underweight and stunting); *P < 0.05, **P < 0.01, ***P < 0.001

^b^Length measurements at birth were available from *n* = 76 participants (HIV-exposed: *n* = 29; HIV-unexposed: *n* = 47; Low gestational mid-upper arm circumference: *n* = 28; High gestational mid-upper arm circumference: *n* = 48)

**Table 5:** Multiple regression analysis of infant weight-for-age and length-for-age z-scores from birth to 6-months using mixed models

Covariate^a^	Weight-for-age z-score			Length-for-age z-score		
	β	*P*	95 % CI	β	*P*	95 % CI
Infant age, months	-0.02	0.091	-0.05, 0.00	-0.13	<0.001	-0.18, -0.09
Infant is female	-1.11	<0.001	-1.44, -0.79	-0.10	<0.001	-1.36, -0.63
Maternal parity	-0.01	0.892	-0.10, 0.09	-0.08	0.141	-0.19, 0.03
Gestational mid-upper arm circumference, cm	0.11	<0.001	0.05, 0.17	0.11	0.001	0.05, 0.18
Mother was formally educated	-0.12	0.629	-0.60, 0.36	-0.03	0.904	-0.57, 0.50
Mother is HIV-positive	-0.65	<0.001	-1.00, -0.31	-0.49	0.012	-0.88, -0.11
Household has electricity	0.40	0.166	-0.17, 0.96	0.46	0.152	-0.17, 1.10
Exclusive breastfeeding duration^b^, wk	-0.02	0.272	-0.06, 0.02	-0.02	0.358	-0.06, 0.02
Breastfeeding score^c^	0.06	0.284	-0.05, 0.17	0.02	0.728	-0.10, 0.14

*HIV* human immunodeficiency virus

^a^Mixed models include all covariates listed

^b^Exclusive breastfeeding according to World Health Organization definition where no food or liquid other than prescribed medications, vitamins/minerals, or oral rehydration fluids given

^c^A study-specific breastfeeding score was evaluated at each visit, and the individual visit scores were summed to provide an overall breastfeeding score. Infant feeding practices were scored as: 0 = no breastfeeding; 1 = partial breastfeeding (defined as breast milk plus other foods and/or liquids); 2 = predominant breastfeeding (defined as breast milk plus locally-prepared non-prescription anti-colic/anti-gripe water); 3 = exclusive breastfeeding. The overall breastfeeding score summarized infant feeding practices over the one to 6-month period, with scores ranging from 0 to 12. In this cohort, the mean breastfeeding score (±standard deviation) was 6.7 ± 2.3

## Discussion

Many studies have demonstrated an association between maternal nutritional status and birth outcomes [15, 16], however there is limited data extending this link to infant growth outcomes. In this prospective cohort of mothers and infants living in northwestern Tanzania, maternal nutritional status defined by gestational MUAC was not only associated with poorer birth anthropometrics, but also ongoing suboptimal infant growth up to 6-months of age. HIV-exposed infants were at greater risk of poorer growth outcomes in this study, despite earlier antenatal care and comparatively better infant feeding practices (although exclusive breastfeeding duration in both groups fell short of WHO recommendations). This provides evidence that HIV-exposed, uninfected infants have poorer birth and growth outcomes in this rural and semi-rural setting. Poorer growth patterns among HIV-infected children are well-documented and several studies have observed differences in growth outcomes between HIV-exposed, uninfected and HIV-unexposed infants in sub-Saharan Africa [17, 18]; however, there is also evidence suggesting that postnatal growth is similar between HIV-exposed, uninfected and HIV-uninfected children, or that differences between groups are small and transient [19, 20].

Among 1005 HIV-positive Malawian women living in an urban setting, MUAC measured at the study enrollment in pregnancy was positively associated with birth weight, and the risk of experiencing a LBW delivery decreased significantly with increasing maternal gestational MUAC [21]. In 1002 HIV-positive pregnant Tanzanian women residing in the capital city of Dar es Salaam, women with MUAC values in the highest quartile gave birth to infants with higher mean birth weights compared to women with MUAC in the lowest quartile, but no association with LBW was observed [22]. The current study is consistent with these observations, and extends these findings by demonstrating this association in a non-urban setting, and uniquely, that gestational MUAC is associated with early infancy anthropometrics and growth up to 6-months of age. While many factors occurring after birth can positively or negatively alter growth trajectories, understanding gestational influences indicates appropriate targets (e.g. women) and timing (e.g. during pregnancy) to prevent negative infant growth outcomes.

A strong and urgent call for POCT to improve health has been made from resource-restricted regions [23]. The ideal POCT has been described according to the ASSURED criteria (developed at the 2003 WHO Special Programme for Research and Training in Tropical Diseases) as being affordable, sensitive, specific, user-friendly, robust and rapid, equipment-free (or requiring minimal equipment), and deliverable [24, 25]. Gestational MUAC appears to be an ideal POCT candidate, requiring a simple tape measure and minimal assessor training. In the current study, a gestational MUAC of ≤25.5 cm (corresponding to the lowest MUAC tertile) was associated with poorer birth and infant growth outcomes; however, further research is needed to define population and disease-specific gestational MUAC reference values. Sub-Saharan African and Asian studies have reported associations with MUAC cutoffs of 22 to 27.6 cm and adverse pregnancy outcomes (LBW, intrauterine growth restriction, preterm delivery) [10, 26–32]. In supplementary feeding programs, 18.5 to 23 cm has been used to identify pregnant women for eligibility [10]. Wasting was defined as <22 cm in a large urban-based mixed-HIV status cohort of pregnant Tanzanians (*n* = 13,760), and found to be more prevalent among HIV-positive women [33]. Since these data were from the pre-ART era, it is difficult to make direct comparisons with more recent studies.

Since many women in a rural and semi-rural setting may never attend an antenatal clinic, POCT with gestational MUAC may be possible to roll-out even at the village level. In addition, many women attend only a single antenatal visit and deliver at home and this suggests that interventions to prevent negative fetal and infant consequences should begin during pregnancy and perhaps a gestational MUAC cut-off on the more conservative upper end is needed. While identification of higher risk pregnancies is a key step in improving fetal and infant care, an ongoing challenge remains in selecting and implementing the most appropriate interventions for these women.

While maternal HIV care continues to improve and important reductions in maternal HIV transmission have been achieved in sub-Saharan Africa, the findings of this study suggest that HIV-exposed, uninfected infants living in this setting still have poorer growth outcomes compared to HIV-unexposed infants. This study was not designed to investigate the impact of ART on growth among HIV-exposed infants and nevirapine adherence was not ascertained. Further research is needed to understand the dual impact of maternal HIV and *in utero* ART-exposure according to various ART regimens on infant growth outcomes. In this cohort, maternal HIV-seropositivity was associated with reduced birth weight and poorer infant growth through 6-months in adjusted analyses. These findings are important to consider since standard antenatal screening performed at this clinic would be unlikely to identify HIV-positive women as being at greater nutritional risk based on mean anthropometric measurements, a finding similar to urban cohorts in Tanzania [33] and Zimbabwe [34]. The birth weight data from this study are consistent with a range of findings from this region [35–37], and also contributes evidence that maternal HIV status is a risk factor for shorter birth length in rural and semi-rural Tanzania. Furthermore, longitudinal data indicate HIV-exposed infants continued to experience less favorable growth anthropometrics as has been observed in other sub-Saharan African cohorts [38], although these findings are not universal [17, 18, 39]. Differences in growth trajectories may be linked to urban-rural differences and a variety of biological and social factors including suboptimal infant feeding practices and maternal illness or death. It is notable that in the present study, HIV-positive mothers exclusively breastfed for a significantly longer duration than HIV-negative mothers and this may partially explain the counterintuitive direction of association between exclusive breastfeeding and infant growth. Nonetheless, the average duration of exclusive breastfeeding in both groups was far shorter than the WHO-recommended 6 months, and comparison to other studies is difficult since the term “exclusive breastfeeding” is not universally defined and evaluated in the same manner.

People living in rural and semi-rural areas account for a substantial proportion of the population in many sub-Saharan African countries, but conducting research in these settings has noteworthy challenges. The proportion of preterm deliveries observed in this study was high (approximately 30 %) and although comparable with other Tanzanian reports [40, 41], inaccuracies in gestational age estimation without ultrasound technology may have resulted in misclassification as health workers must instead rely on unreliable methods such as patient recall of last menstruation and fundal height [42]. During the course of this study, Kisesa Health Centre faced chronic and variable healthcare supply shortages. This may have deterred women from traveling to the clinic to receive antenatal and infant care, and limits the generalizability of these findings to women willing and able to attend clinic visits. It should also be noted that pregnant women in this study were only tested for HIV once, as part of standard antenatal care at the host clinic, and it is possible that some HIV-negative women may have seroconverted after being tested.

As is common in rural Africa, some participants were difficult to contact without traceable addresses or telephone numbers. To minimize attrition, participants consented to home visits by a study team member if they missed an appointment, a strategy that proved invaluable in minimizing losses to follow-up. Finally, despite provision of incidental clinic delivery costs and transportation to women who chose to deliver at this antenatal clinic, complete birth anthropometric data within 72 h were unavailable for 24 % of infants - a proportion that represents a study success given that approximately 50 % of births typically occur at a place other than a health facility in this region [11].

## Conclusions

Low infant birth weight is an important risk factor for early infancy morbidity and mortality [2], making prevention of fetal and infant growth impairment a key goal. Gestational MUAC was able to identify women whose infants experienced poorer birth and growth anthropometrics. While identifying at-risk mothers is only the first step towards improving pregnancy and infant outcomes, evaluation of gestational MUAC as a POCT and establishing appropriate reference values is needed. Maternal health based on standard clinical (e.g. Hb concentration) and gestational anthropometric indicators did not differ according to maternal HIV status; however, HIV-exposed, uninfected infants still fared worse in this rural and semi-rural setting and maternal HIV-seropositivity alone remains was a risk factor for poorer fetal and infant growth.

## Abbreviations

ANOVA: Analysis of variance
ART: Antiretroviral
BMI: Body mass index
CI: Confidence interval
Hb: Hemoglobin
HIV: Human immunodeficiency infection
LAZ: Length-for-age z-scores
LBW: Low birth weight
MUAC: Mid-upper arm circumference
PMTCT: Prevention of mother to child transmission
POCT: Point-of-care testing
SD: Standard deviation
TSF: Triceps skinfold thickness
WAZ: Weight-for-age z-scores
WHO: World Health Organization
